# Myosin Va: Capturing cAMP for synaptic plasticity

**DOI:** 10.3389/fphys.2023.1342994

**Published:** 2024-01-04

**Authors:** Rüdiger Rudolf

**Affiliations:** ^1^ Center for Mass Spectrometry and Optical Spectroscopy (CeMOS), Mannheim University of Applied Sciences, Mannheim, Germany; ^2^ Interdisciplinary Center for Neurosciences, Heidelberg University, Heidelberg, Germany; ^3^ Mannheim Center for Translational Neurosciences, Heidelberg University, Mannheim, Germany

**Keywords:** acetylcholine receptors, AKAP, cAMP, microdomain, myosin Va, neuromuscular junction, PKA, synaptic plasticity

## Abstract

The plus-end directed actin-dependent motor protein, myosin Va, is of particular relevance for outward vesicular protein trafficking and for restraining specific cargo vesicles within the actin cortex. The latter is a preferred site of cAMP production, and the specificity of cAMP signaling is largely mediated through the formation of microdomains that spatially couple localized metabotropic receptor activity and cAMP production to selected effectors and downstream targets. This review summarizes the core literature on the role of myosin Va for the creation of such a cAMP microdomain at the mammalian nerve–muscle synapse that serves the activity-dependent recycling of nicotinic acetylcholine receptors (nAChRs)—a principal ligand-gated ion channel which is imperative for voluntary muscle contraction. It is discussed that i) the nerve–muscle synapse is a site with a unique actin-dependent microstructure, ii) myosin Va and protein kinase A regulatory subunit Iα as well as nAChR and its constitutive binding partner, rapsyn, colocalize in endocytic/recycling vesicles near the postsynaptic membrane, and iii) impairment of myosin Va or displacement of protein kinase A regulatory subunit Iα leads to the loss of nAChR stability. Regulation of this signaling process and underlying basic pieces of machinery were covered in previous articles, to which the present review refers.

## 1 Main text

### 1.1 The cooperative/capture model for myosin Va

Myosin Va is an actin-dependent motor protein with particular relevance in the context of outward transport—exocytosis, and recycling—of vesicular carriers in different cell types. Along with myosin Vb and myosin Vc, myosin Va is one of three myosin V isoforms found in mammals (Reck-Peterson et al., 2000). All three are composed of a heavy chain dimer and a variable number of light chains. The heavy chains exhibit an actin-binding N-terminal motor domain, followed by a regulatory neck region containing calmodulin-binding sites, a stalk region mediating dimerization of the myosin V heavy chains, and a C-terminal cargo-binding domain (Reck-Peterson et al., 2000). Although all myosin-V isoforms are processive and plus-end-directed actin-dependent motor proteins, they appear to carry different cargo. At least partially, this is due to differential interaction with Rab-GTPases and further adaptor proteins (Wong and Weisman, 2021). Concerning myosin Va, a lack of its protein function in humans leads to Elejalde (Sanal et al., 2000) or Griscelli syndrome (Pastural et al., 1997), which is characterized by a loss of skin and hair pigmentation, immune deficiency, and severe neurological symptoms, including seizures and muscle weakness (Griscelli et al., 1978). A very similar phenotype is observed in *dilute lethal* mice, a mutant mouse strain that carries a dominant-negative version of myosin Va (Mercer et al., 1990). Homozygous *dilute lethal* mice exhibit silvery-gray hair and die with seizures at roughly 3 weeks of age (Mercer et al., 1990).

Concerning the reasons for the pigmentation deficiency found in Griscelli patients and *dilute lethal* mice, the cooperative/capture model for the function of myosin Va was proposed in a seminal study using pigment-transporting melanocytes (Wu et al., 1998). The work described fast anterograde and retrograde microtubule-dependent transport of pigment granules (melanosomes) from the center to the periphery of melanocytes and back. In wild-type cells, most pigment granules were captured upon their arrival in the actin cortex near the cell membrane, leading to their accumulation in the cell tips. Conversely, in pigment cells from *dilute lethal* mice, melanosomes were unrestrained in the actin cortex. Therefore, they accumulated in the region with the highest microtubule density, that is, the cell center. In the absence of microtubules, the movement of melanosomes was almost null in *dilute lethal* melanocytes, whereas a motility component with a speed of 0.1–0.4 μm/s was visible in wild-type cells. The expression of a dominant-negative myosin Va C-terminal domain (MCLT) in wild-type melanocytes created a “*dilute*”-like accumulation of melanosomes in the cell center. Altogether, these data argued for the cooperation of microtubule and actin-dependent transport and for the critical role of myosin Va in capturing melanosomes in the actin-rich cell periphery.

Thus, myosin Va is critical for the trafficking and selective subcellular positioning of melanosomes. However, myosin Va was also found to transport other organelles and subcellular structures. These include secretory granules in neuroendocrine cells (Rudolf et al., 2003), ribonucleoprotein complexes in dendrites and axons of neurons (Calliari et al., 2014), insulin-regulated glucose transporter type 4-containing recycling endosomes at postprandial states (Sun et al., 2014), exocytic and Ca^2+^-regulated asymmetric vesicle transport during chemotactic axon growth (Wada et al., 2016), and the F-actin disassembly regulator, MICAL1 (microtubule associated monooxygenase, calponin, and LIM domain containing 1), at the midbody during cytokinesis (Niu et al., 2020). In addition, the severe neurological phenotype of Griscelli patients and *dilute lethal* mice hinted at a putative function of myosin Va in motor control or the neuromuscular apparatus.

### 1.2 Activity-dependent regulation of nAChR turnover in the actin-rich postsynapse

**FIGURE 1:**
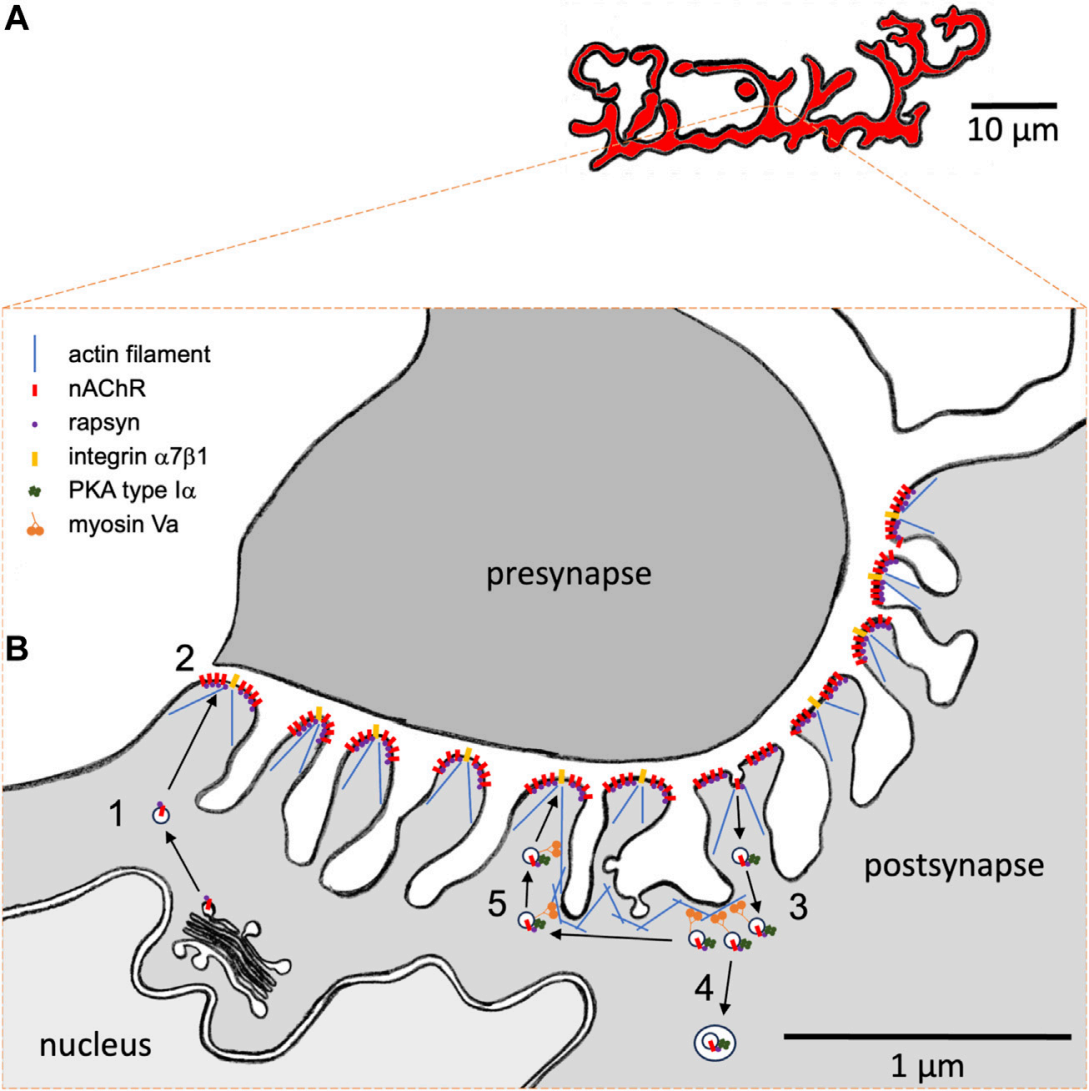
Hypothetical model of the role of myosin Va in the subsynaptic trafficking of nicotinic acetylcholine receptors (nAChR) at the mammalian neuromuscular junction (NMJ). **(A)** Representative macroscopic “pretzel”-like morphology of a healthy adult mouse NMJ. nAChRs are highly concentrated in band-like arrays with a width of 1–3 µm. NMJs usually span over a few tens of micrometers. Each muscle fiber contains one single NMJ, which usually resides roughly in the center of a muscle fiber. Motor neuron presynapse and muscle fiber postsynapse normally show a perfect match on the band-like arrays. **(B)** High-resolution view on a cross-section through an individual synaptic band. Motor neuron presynapse, top; postsynapse, bottom. The muscle fiber postsynaptic membrane shows deep junctional folds. nAChRs and integrin α7β1 are located on the crests of folds. Furthermore, dystrophin and utrophin are concentrated at the NMJ (not shown). The actin cytoskeleton is crucial for fold occurrence. Subsynaptic nuclei and secretory apparatus with ER and Golgi apparatus as well as numerous vesicular structures can be regularly observed near the postsynaptic membrane. Upon biogenesis and post-translational glycosylation, nAChRs are transported to the postsynaptic membrane in the presence of rapsyn. (1) Upon biogenesis and post-translational glycosylation, nAChRs are transported to the postsynaptic membrane in the presence of rapsyn. (2) At the membrane, agrin-dependent nAChR clustering occurs in the presence of rapsyn. (3) Endocytosis and accumulation of nAChR-containing carriers is observed next to the postsynaptic membrane. Depending on synaptic/fiber activity status, nAChRs are then either (4) degraded using autophagic processes or (5) recycled. Activity-dependent recycling of nAChRs needs myosin Va and rapsyn-mediated anchoring of PKA RIα on nAChR-recycling vesicles. Presumably, myosin Va captures these vesicles near the postsynaptic membrane in an activity-dependent cAMP microdomain and is therefore intrinsically critical for the recycling itself.

In vertebrates, neuromuscular transmission occurs at special synapses, called neuromuscular junctions (NMJs), between cholinergic lower motor neurons and skeletal muscle fibers. Their postsynaptic apparatus is characterized by a complex band-like, often “pretzel”-shaped, high-density arrangement (Figure 1A) of transmembrane nicotinic acetylcholine receptors (nAChRs), which are juxtaposed to the presynaptic membrane and are essential for neuromuscular transmission. Each band has a width of roughly 1–3 µm, and their ultrastructural analysis showed that the postsynaptic membrane of mammalian NMJs is usually deeply invaginated, forming junctional folds with a depth of a few hundred nanometers (Palade and Palay, 1954; Reger, 1955) (Figure 1B). Within a fold, nAChRs are mostly found at the crests, whereas other proteins, such as voltage-gated channels necessary for action potential generation, are located in the troughs of the folds (Flucher and Daniels, 1989).

Notably, postsynaptic nAChRs exhibit a remarkable metabolic regulation: whereas a long nAChR lifetime with a t_1/2_ of 10–12 days is typical for innervated NMJs, their t_1/2_ is shortened to only 1–2 days under several denervating or disease conditions (Fambrough et al., 1973; Stanley and Drachman, 1981; Ramsay et al., 1992; Strack et al., 2011; Strack et al., 2015). Similar to other transmembrane and secretory proteins, nAChR biogenesis involves co-translational import into the rough endoplasmic reticulum at subsynaptic nuclei, followed by subunit assembly, glycosylation in the Golgi apparatus, and exocytic transport to the postsynaptic membrane (for review, see Sanes and Lichtman, 1999; Tintignac et al., 2015; Li et al., 2018) (process 1 in Figure 1B). During this transport (Marchand et al., 2002) and likely during all subsequent stages (Chen et al., 2016), nAChRs are accompanied by a 43 kDa scaffold protein (Frail et al., 1988), termed rapsyn. Rapsyn is also involved in the clustering of nAChRs at the postsynaptic membrane (process 2 in Figure 1B), a process that is induced by the neuronal release of agrin (McMahan and Wallace, 1989), which activates a signal cascade *via* low-density lipoprotein receptor-related protein 4 (LRP4) (Kim et al., 2008; Zhang et al., 2008), muscle-specific kinase (MuSK) (Valenzuela et al., 1995; DeChiara et al., 1996), and downstream of tyrosine kinases-7 (Dok7) (Okada et al., 2006) proteins (for review, see Tintignac et al., 2015; Li et al., 2018). At the postsynaptic membrane, nAChRs fulfill their function as ligand-gated ion channels. *In situ* microscopy of muscles labeled with fluorescent α-bungarotoxin, which is a highly specific marker of nAChR, showed the occurrence of endocytosis of nAChR (process 3 in Figure 1B), with high amounts of nAChR-endocytic carriers under denervated/disease conditions and a low number in normal muscle (Akaaboune et al., 1999). A modification of the fluorescent α-bungarotoxin assay revealed that endocytosed nAChRs might then be either degraded (process 4 in Figure 1B) or recycled (process 5 in Figure 1B), again depending on the activity/innervation status of the synapses (Bruneau et al., 2005; Bruneau and Akaaboune, 2006; Khan et al., 2014). The clear regulatory aspect of nAChR turnover suggested the involvement of extrinsic factors that would act upon a putative endocytosis/recycling/degradation machinery.

The NMJ is a site with a particularly well-developed and organized microtubule network (Rahkila et al., 1997; Ralston et al., 1999), actin cortex (Bloch and Hall, 1983; Bloch, 1986; Cartaud et al., 2011), and intense vesicular trafficking (Fumagalli et al., 1982; Akaaboune et al., 1999; Khan et al., 2014). Already in the course of NMJ development, during which nAChR clusters are converted in the perinatal period from simple, plaque-like structures to fenestrated plaques and finally to a highly complex band-like array (pretzel-shape) (Slater, 1982; Marques et al., 2000), the actin cortex near the nAChR clusters is remodeled by several proteins, forming the so-called podosomes (Proszynski et al., 2009; for review, see Bernadzki et al., 2014). Some of these proteins link to actin and/or the dystrophin/utrophin glycoprotein complex. Both dystrophin and its sibling, utrophin, are major actin-coordinating proteins in skeletal muscle, and both are highly abundant in the NMJ (Banks et al., 2003). The lack of either of these leads to massive alterations of NMJ morphology (Deconinck et al., 1997; for review, see Dobbins et al., 2006) and a loss of junctional folds, suggesting that the actin cytoskeleton is important for fold formation and/or maintenance. Along these lines, super-resolution microscopy revealed that NMJ-specific integrin α7β1 is located, similar to nAChR, at the crest of junctional folds. However, whereas nAChRs were suggested to be localized more on the lateral crest border zones, integrins were found at the crest centers (York and Zheng, 2017) (Figure 1B). Integrins, just like dystrophin and utrophin, usually connect to the actin cytoskeleton. This further corroborates the eminent role of the actin cytoskeleton in shaping the postsynaptic apparatus with putative functional implications. Agrin-induced clustering of nAChR was also connoted with raft formation and actin cytoskeleton reorganization (Cartaud et al., 2011), a process that was mediated by the actin remodeling enzyme, coronin 6 (Chen et al., 2014).

### 1.3 Role of myosin Va in nAChR turnover

Using several approaches, the following condition was addressed: if the actin cytoskeleton near the NMJ would also be critical for nAChR trafficking. As outlined below, these studies included immunofluorescence, the use of myosin Va-deficient *dilute lethal* mice, transient overexpression of dominant-negative versions of myosin Va (MCLT and MCST), and knockdown of myosin Va. In healthy muscle, myosin Va immunofluorescence signals were clearly enriched in the NMJ region, and the accumulation increased strongly from P1 to P16 and then stayed constant during adulthood (Röder et al., 2008). Next, a comparison of healthy and *dilute lethal* muscles showed that NMJ maturation was deficient in the diseased muscles. Whereas in the wild type, the typical transition from plaque to perforated plaque to pretzel-shape occurred during the first three postnatal weeks, the *dilute lethal* NMJs went from plaque to disintegration (Röder et al., 2008). Whereas in normal mice, the first postnatal weeks also featured a continuous increase in the half-life of nAChRs to reach its typical value of 10–12 days (Yampolsky et al., 2010b), a metabolic nAChR stabilization was completely lacking in *dilute lethal* mice (Yampolsky et al., 2010a). To investigate the putative role of myosin Va in stabilizing nAChRs in adult muscle, a sequential nAChR labeling approach was coupled to transient downregulation of myosin Va expression. This revealed that acute myosin Va knockdown in adult wild-type mouse muscle would lead to a massively decreased stability of nAChR (Röder et al., 2008). In summary, these data pointed to a specific role of myosin Va at the NMJ and, in particular, in stabilizing nAChRs in a process that was implemented during the phase of postnatal NMJ maturation from plaque to pretzel.

Next, to address the mechanism behind this putative myosin Va-dependent stabilization, three findings were of importance (Röder et al., 2008): first, in live mouse muscle, myosin Va colocalized and co-precipitated with endocytosed, α-bungarotoxin-positive nAChRs. Second, the NMJ size decreased upon overexpression of a myosin Va-dominant-negative mutant. Third, upon myosin Va-inhibition, the number of endocytic nAChR carriers was massively increased, and these puncta were not confined close to the postsynaptic membrane (as in untreated muscle) but were distributed throughout the entire fiber diameter. These findings hinted at the cooperative/capture model (see above) and suggested that myosin Va might be crucial to restraining endocytic/recycling vesicles carrying nAChRs near the plasma membrane (process 5 in Figure 1B).

### 1.4 A cAMP microdomain for nAChR turnover regulation

Previous reports proposed that the local production of 3′,5′-cyclic adenosine monophosphate (cAMP) upon neuronal release of the signal molecule, α-calcitonin gene-related peptide (CGRP), would enhance the stabilization of nAChR clusters at the postsynaptic membrane during embryogenesis (Lanuza et al., 2002). Furthermore, it was found that cAMP was critical for nAChR turnover regulation in the adult mouse (Shyng et al., 1991), and that a specific isoform of the regulatory subunit of protein kinase A (PKA), namely, PKA RIα, was highly enriched in a punctiform pattern next to the NMJ (Barradeau et al., 2001; Barradeau et al., 2002). In the field of signal transduction, it is a widely accepted concept that the specificity of second messenger signaling is mediated by microdomain formation, whereby essential components of a signaling pathway, that is, from transmembrane receptor over second messenger production to effector activity, are spatially restricted and segregated from neighboring microdomains (Zaccolo, 2011; Filadi and Pozzan, 2015; Omar and Scott, 2020). Consequently, whether myosin Va might be critical for nAChR stabilization by capturing endocytosed nAChRs within such a putative cAMP/PKA microdomain near the NMJ postsynaptic membrane was addressed. These studies revealed that in adult wild-type muscle, PKA type RIα, but not type RII, accumulated in numerous puncta near the NMJ. Furthermore, these puncta were also positive for endocytic nAChR and myosin Va (Röder et al., 2010). The knockdown of myosin Va massively reduced this PKA RIα enrichment, NMJ size, and nAChR stability (Röder et al., 2010). Next, treatment with CGRP led to an acute rise of cAMP in the region of PKA RIα puncta, and this effect was lost upon myosin Va knockdown (Röder et al., 2010). Altogether, these data were consistent with the existence and functional relevance of a protein complex composed of myosin Va, PKA RIα, and nAChR on endocytic/recycling vesicles. Furthermore, the studies indicated that this protein complex had to be located in a subsynaptic and active cAMP microdomain to allow recycling and a longer lifetime of nAChRs.

A-kinase anchoring proteins (AKAPs) are key to organizing such cAMP/PKA microdomains (Omar and Scott, 2020). AKAPs are characterized by at least three principal molecular features: first, special amphipathic α-helical protein domains mediate the interaction of AKAPs with PKA regulatory subunits. Second, AKAPs exhibit specific subcellular targeting domains that allow anchoring of the PKA to the site of interest. Third, to serve as scaffolding proteins, AKAPs regularly bind other signaling components involved in a given cAMP/PKA-dependent signal pathway. In the search for a potential AKAP candidate at the NMJ, evidence from previous studies pointed to rapsyn as it i) exhibits an amphipathic α-helix; ii) targets the sites of interest, that is, NMJs and nAChR; and iii) was known to exhibit multiple binding sites to further potential signal components (Ramarao et al., 2001; Lee et al., 2008; Lee et al., 2009). Fittingly, molecular modeling corroborated the rapsyn α-helical region as a potential interaction domain to PKA RIα. Furthermore, full-length rapsyn, but not rapsyn lacking the α-helix, co-precipitated with PKA RIα in culture cells (Choi et al., 2012). In addition, an *in vivo*-bimolecular fluorescence complementation assay indicated a direct interaction of rapsyn and PKA RIα in puncta exclusively near the NMJ in a punctiform pattern that was reminiscent of the distribution of PKA RIα from previous reports (Barradeau et al., 2001; Barradeau et al., 2002; Röder et al., 2010; Choi et al., 2012). Overexpression of competitive rapsyn α-helix alone or another RI anchoring disruptor peptide (RIAD) (Carlson et al., 2006) displaced PKA RIα from its normal location next to NMJ (Röder et al., 2010; Choi et al., 2012). Finally, overexpression of rapsyn α-helix or rapsyn lacking its α-helix led to NMJ fragmentation and a significantly reduced nAChR lifetime (Choi et al., 2012). Altogether, these data suggested that rapsyn serves as an AKAP that bridges PKA RIα to nAChRs in endocytic/recycling vesicles. Within this concept, tethering by myosin Va to a subsynaptic cAMP microdomain would regulate nAChR recycling to the postsynaptic membrane and the metabolic lifetime of nAChRs. This putative nAChR recycling apparatus might be tuned by intra- and extracellular signals, where its activation would presumably lead to nAChR recycling (process 5 in Figure 1B) and its inactivation would lead to nAChR degradation (process 4 in Figure 1B) and thus affect the overall nAChR stability.

### 1.5 Conclusion

Until now, a series of functional or molecular factors have been found to modulate nAChR lifetime and stability (for review, see Rudolf and Straka, 2019; Martinez-Pena Y Valenzuela and Akaaboune, 2021), including muscle activity, innervation, PKC, and CaMKII. Furthermore, the basic types of machinery underlying the exocytosis, endocytosis, degradation, and recycling of nAChRs have been further characterized (for review, see Rudolf and Straka, 2019; Martinez-Pena Y Valenzuela and Akaaboune, 2021). As for other cell types and trafficking processes, dedicated small GTPases of the Rab-protein family are critical and show specific distributions at the NMJ (Wild et al., 2016; Wild et al., 2019). However, a plethora of important questions have remained unsolved. For example, how do external and internal regulatory factors tune the nAChR recycling/degradation decision, that is, which are the molecular targets that get switched? Where precisely in or close to the junctional folds do nAChR endocytosis, recycling, and degradation occur, that is, is there a spatial separation of endocytic, recycling, and degradation processes? What is the correlation between nAChR lifetime and endocytosis/recycling, that is, do nAChRs recycle multiple times?

This review proposed a concept, whereby recycling vesicles carrying postsynaptic ligand-gated ion channels are confined to the actin cytoskeleton by myosin V; furthermore, this localization is critical for regulation by second messengers. Although the evidence described here was acquired at NMJs, the general principle might not be exclusive to the peripheral nerve–muscle synapses. Indeed, the myosin Va sibling, myosin Vb, was associated with the activity-dependent recycling of AMPA receptors in hippocampal neurons (Wang et al., 2008). Although it is accepted that such recycling of AMPA receptors is under the control of Ca^2+^, it is debated if this second messenger directly modulates myosin Vb (Wang et al., 2008) or synaptotagmin 1/7 (Sumi and Harada, 2020) to achieve activity-dependent synaptic plasticity. It will be interesting to see how these complementary fields of research will mutually stimulate each other.
